# Two Coselected Distal Mutations in HIV-1 Reverse Transcriptase (RT) Alter Susceptibility to Nonnucleoside RT Inhibitors and Nucleoside Analogs

**DOI:** 10.1128/JVI.00224-19

**Published:** 2019-05-15

**Authors:** Paul L. Boyer, Kevin Melody, Steven J. Smith, Linda L. Dunn, Chris Kline, Douglas K. Fischer, Richa Dwivedi, Pat Clark, Stephen H. Hughes, Zandrea Ambrose

**Affiliations:** a HIV Dynamics and Replication Program, National Cancer Institute, National Institutes of Health, Frederick, Maryland, USA; b Department of Infectious Diseases and Microbiology, University of Pittsburgh Graduate School of Public Health, Pittsburgh, Pennsylvania, USA; c Department of Microbiology and Molecular Genetics, University of Pittsburgh School of Medicine, Pittsburgh, Pennsylvania, USA; d Retroviral Replication Laboratory, Basic Science Program, Leidos Biomedical Research, Inc., Frederick National Laboratory for Cancer Research, Frederick, Maryland, USA; University of Utah

**Keywords:** drug resistance, nucleic acid repositioning, nucleoside analogs, nucleoside inhibitor, nonnucleoside RT inhibitor

## Abstract

Two mutations, G112D and M230I, were selected in the reverse transcriptase (RT) of human immunodeficiency virus type 1 (HIV-1) by a novel nonnucleoside reverse transcriptase inhibitor (NNRTI). G112D is located near the HIV-1 polymerase active site; M230I is located near the hydrophobic region where NNRTIs bind. Thus, M230I could directly interfere with NNRTI binding but G112D could not. Biochemical and virological assays were performed to analyze the effects of these mutations individually and in combination. M230I alone caused a reduction in susceptibility to NNRTIs, while G112D alone did not. The G112D/M230I double mutant was less susceptible to NNRTIs than was M230I alone. In contrast, both mutations affected the ability of RT to incorporate nucleoside analogs. We suggest that the mutations interact with each other via the bound nucleic acid substrate; the nucleic acid forms part of the polymerase active site, which is near G112D. The positioning of the nucleic acid is influenced by its interactions with the “primer grip” region and could be influenced by the M230I mutation.

**IMPORTANCE** Although antiretroviral therapy (ART) is highly successful, drug-resistant variants can arise that blunt the efficacy of ART. New inhibitors that are broadly effective against known drug-resistant variants are needed, although such compounds might select for novel resistance mutations that affect the sensitivity of the virus to other compounds. Compound 13 selects for resistance mutations that differ from traditional NNRTI resistance mutations. These mutations cause increased sensitivity to NRTIs, such as AZT.

## Abstract (precis)

Although antiretroviral therapy (ART) is highly successful, drug-resistant variants can arise that blunt the efficacy of ART. New inhibitors that are broadly effective against known drug-resistant variants are needed, although such compounds might select for novel resistance mutations that affect the sensitivity of the virus to other compounds. Compound 13 selects for resistance mutations that differ from traditional NNRTI resistance mutations. These mutations cause increased sensitivity to NRTIs, such as AZT.

## INTRODUCTION

Nonnucleoside reverse transcriptase inhibitors (NNRTIs), which target the essential viral enzyme reverse transcriptase (RT), have been an integral part of human immunodeficiency virus type 1 (HIV-1) therapy since they were first approved for use in the United States in 1996. RT contains a DNA polymerase domain (which can copy either a DNA template or an RNA template) and an RNase H domain, which degrades the RNA strand when it is in an RNA:DNA heteroduplex. NNRTIs bind in a hydrophobic region approximately 10 Å from the polymerase active site, creating a binding pocket that does not exist in the absence of a bound NNRTI. NNRTI binding distorts RT, pushing the 3′ end of the primer away from the active site, preventing polymerization (1). This is in contrast to nucleoside RT inhibitors (NRTIs), which are nucleoside analogs that lack the 3′ hydroxyl group. When NRTIs are incorporated into the nascent viral DNA strand by RT, they terminate DNA synthesis. As with all HIV-1 inhibitors, drug resistance mutations can be selected that decrease or eliminate the effectiveness of both NNRTIs and NRTIs. Some drug resistance mutations confer cross-resistance to multiple drugs in the same class, which is often the case for NNRTIs (2).

The most recently FDA-approved NNRTI, rilpivirine (RPV), is a diarylpyrimidine that has structural flexibility, allowing it to adapt to changes in RT structure and maintain efficacy against many of the common resistance mutations selected by more rigid NNRTIs, such as efavirenz (EFV) and nevirapine (NVP) (3–5). In cell culture studies, it was more difficult to select for resistance to RPV than for resistance to EFV and NVP; high levels of RPV resistance usually required multiple mutations (5). RPV and EFV were shown to have similar efficacies in two phase III clinical trials, but RPV had better tolerability and selected for different resistance mutations (6, 7). In those trials, the viruses in participants who failed RPV-containing therapies had the RT mutations L100I, K101E, K103N, V108I, E138K/R, Y181C, and/or M230L. E138K was the mutation present at the highest frequency (8). Its ability to retain efficacy against several common NNRTI resistance mutations and a favorable safety profile make RPV an attractive NNRTI. However, RPV resistance can be selected both in cell cultures and in patients, and mutations exist that reduce the efficacy of all FDA-approved NNRTIs (4, 5, 9, 10).

**FIG 1:**
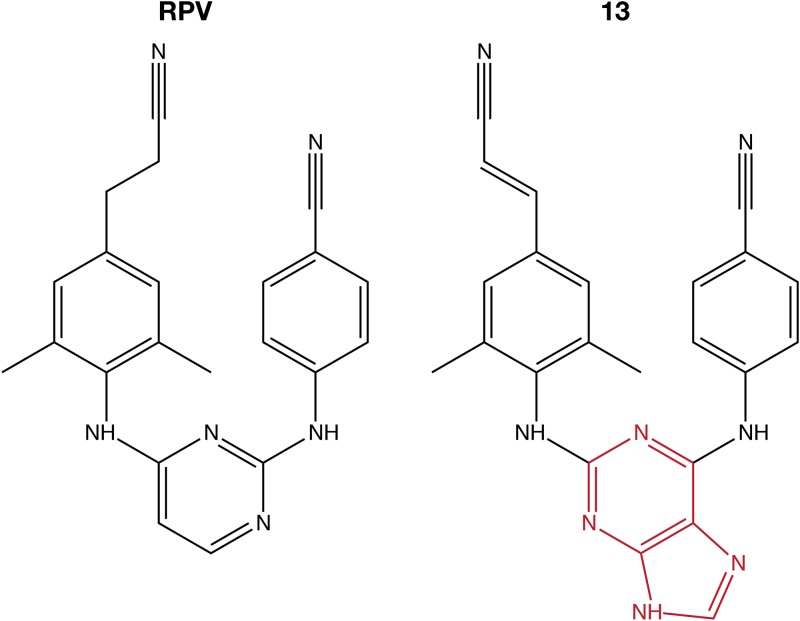
Chemical structures of RPV and compound 13.

We developed RPV analogs that have changes in the pyrimidine and 4-amino-benzonitrile moieties. These changes were intended to alter the spectrum of mutations that confer resistance to the compound (11). The acrylonitrile ring moiety was not changed in any of our derivatives because this moiety interacts with a hydrophobic pocket and appears to be important for RPV binding (3, 4, 12). One analog, compound 13 (Fig. 1), differs from RPV by replacement of the central pyrimidine ring with a 2,6 purine ring system in a “flipped” conformation that may allow better interactions with the hydrophobic region; however, compound 13 does have similar side groups. Compound 13 is similar to RPV in terms of its therapeutic index and its antiretroviral efficacy against a panel of HIV-1 mutants containing RT mutations, including L100I, K103N, V106A, E138K, Y181C, Y188L, H221Y, and K103N/Y181C (11). Here we report that HIV-1 replicating in the presence of compound 13 coselected two mutations in RT (G112D and M230I), which were analyzed using biochemical and virological assays to understand their role(s) in drug susceptibility and their effects on HIV-1 replication. Despite the fact that these two mutations were selected with an NNRTI, the mutations affected the susceptibility of the virus (and RT) to inhibition by both NNRTIs and NRTIs. We propose that the selected mutations interact through the bound nucleic acid, which provides insight into the behavior of RT and into how they affect the susceptibility of HIV-1 to both NNRTIs and NRTIs.

## RESULTS

### Compound 13 selects G112D and M230I in HIV-1 RT.

**FIG 2:**
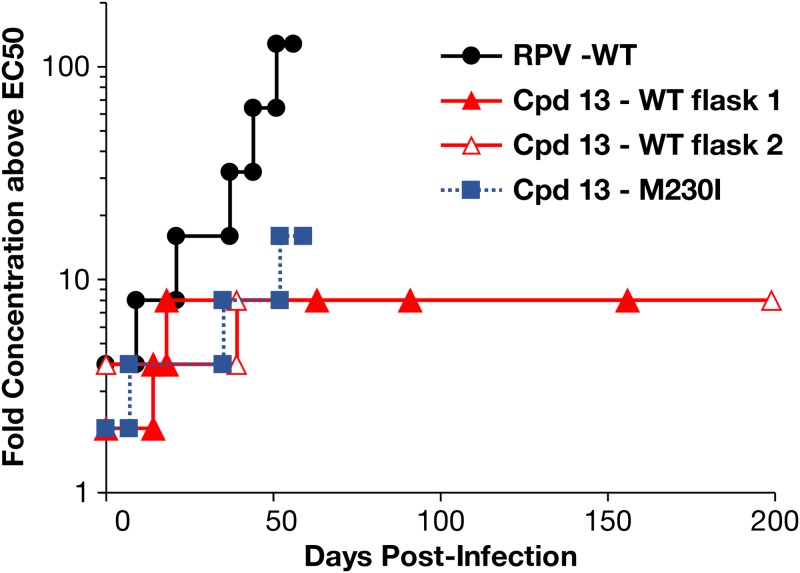
Selection of RT mutations. HuT-R5 cells were infected with replication-competent WT HIV-1 or HIV-1 with the mutation M230I in the RT. The cells were cultured in the presence of increasing concentrations of RPV or compound (Cpd) 13.

We have previously described an RPV analog, compound 13, that was developed to inhibit both wild-type (WT) HIV-1 and the common NNRTI-resistant mutants (11, 13) (Fig. 1). The compound potently inhibited the broad range of NNRTI-resistant mutants that we tested. To determine if other mutations would reduce the potency of compound 13, we used it to select mutations in HIV-1. HuT-R5 cells were infected with HIV-1 in 2 separate flasks in the presence of a concentration of compound 13 that was 2- or 4-fold above the 50% effective concentration (EC_50_), and the virus was passaged in the presence of increasing concentrations of compound 13 or RPV, which was used in a parallel selection (Fig. 2). The previously described RPV-associated mutation K101E (5) was detected in the RPV selected cultures by sequencing viral RNA after 56 days, when the drug was present at 128-fold above the EC_50_. In contrast, cytopathic effects (CPE) were not observed in cultures containing compound 13 for >90 days, although the concentration of compound 13 was 8-fold above the EC_50_ (Fig. 2). Thus, there were differences in the response of HIV to selection with compound 13 and RPV both in the change in susceptibility (8-fold versus 128-fold) and in the time it took to observe CPE. When CPE were finally observed, bulk sequencing of viral RNA and DNA from both compound 13 cultures consistently identified RT mutations G112D and M230I; G112D was detected first in one of the cultures (Table 1). RNA and DNA molecules were cloned and sequenced, and the results showed that G112D and M230I were linked on the same genomes, suggesting that both mutations contributed to the observed loss of potency of the compound.

**TABLE 1:** HIV-1 RT mutations detected after selection in the presence of compound 13

Virus	Days of selection	Nucleic acid sequenced	Amino acids sequenced	RT mutations^*a*^
WT, flask 1	63	RNA (bulk PCR)	1–250	K64*, S69C, G112D
	91	RNA (bulk PCR)	1–250	G112D, M230I
	156	RNA (bulk PCR)	1–140, 228–450	G112D, M230I, V381L
		DNA (bulk PCR)	1–120, 210–560	G112D, M230I
		RNA (clone)	1–560	G112D, M230I
		RNA (clone)	1–560	V10I, G112D, K126E, M230I
		RNA (clone)	1–560	K43R, E53G, K281*, E297G, A360V, P433L
		DNA (clone)	1–560	K101E, M230I, L391P
WT, flask 2	199	RNA (bulk PCR)	1–560	G112D, M230I
		DNA (bulk PCR)	1–560	G112D, M230I
		RNA (clone)	1–560	G112D, M230I
		RNA (clone)	1–560	G112D, M230I
		RNA (clone)	1–560	G112D, M230I, L517F^FS^
		RNA (clone)	1–560	G112D, M230I, D250V, T403I
		DNA (clone)	1–560	G112D, M230I
		DNA (clone)	1–560	G112D, M230I, K331E
		DNA (clone)	1–560	P55L, G112D, M230I, R448G
M230I mutant	59	RNA (bulk PCR)	1–470	G112D, G196R, M230I

a *, stop codon; FS, frameshift.

M230I was introduced into a molecular clone and selection was performed with compound 13 to determine whether G112D would be selected again. After 59 days of infection, viral RNA was isolated and the G112D and G196R mutations were detected in RT (Table 1). These results show that G112D was selected in three independent cultures with M230I, suggesting that both amino acid substitutions provide an advantage for HIV-1 replication in the presence of compound 13.

**FIG 3:**
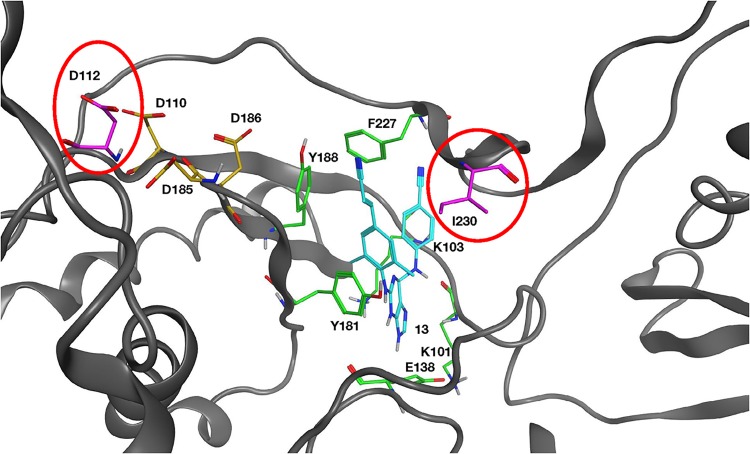
Overview of the HIV-1 RT structure near the polymerase active site. Compound 13 (cyan) is bound at the NNRTI binding pocket. Residues that comprise the NNRTI binding pocket and commonly undergo resistance mutations are K101, K103, E138, Y181, Y188, and F227 and are depicted in green. The three aspartic acid residues (D110, D185, and D186) that bind metal ions at the polymerase active site are shown in gold. The mutated G112D and M230I are modeled into the structure and are shown in magenta and circled red. The RT ribbon backbone is dark gray, while the nitrogen atoms in the residues are blue and the oxygen and hydrogen atoms are red and light gray, respectively.

Figure 3 shows the location of these mutated amino acids relative to the polymerase active site and the hydrophobic NNRTI binding region. M230 is part of the “primer grip,” which interacts with the primer strand of the bound nucleic acid. However, M230 is also near the NNRTI binding site, and the M230I mutation is in a position to interact directly with compound 13. Position 112 is located near the polymerase active site, but it is on the opposite side from both M230I and a bound NNRTI. G112D is located approximately 10.0 Å from the NNRTI binding region and is not in a position where it could interact directly with a bound NNRTI or with M230I (Fig. 3).

### G112D alone does not reduce susceptibility to NNRTIs but does reduce susceptibility to NNRTIs in combination with M230I.

RTs containing G112D, M230I, or the double mutation G112D/M230I were introduced into a molecular clone of HIV-1, and their effects on the potencies of compound 13, RPV, EFV, etravirine (ETR), and NVP were measured (Table 2). G112D alone did not alter the susceptibility of the virus to any of these NNRTIs; in contrast, M230I caused a 7- to 11-fold decrease in susceptibility. The addition of the G112D mutation to M230I increased the EC_50_ values for compound 13 and RPV 3-fold, for EFV and ETR 2-fold, and for NVP 1.5-fold compared to the value with M230I alone.

**TABLE 2:** WT and mutant HIV-1 susceptibility to NNRTIs

Virus	EC_50_^*a*^ (fold change above WT)				
	Compound 13	RPV	EFV	ETR	NVP
WT	1 ± 0.2 (1)	0.4 ± 0.1 (1)	11 ± 1 (1)	0.7 ± 0.08 (1)	79 ± 18 (1)
G112D	1 ± 0.3 (1)	0.7 ± 0.04 (2)	13 ± 2 (1)	0.8 ± 0.1 (1)	52 ± 11 (1)
M230I	**9 ± 1 (7)**	**4 ± 0.6 (10)**	**89 ± 9 (8)**	**5 ± 0.2 (7)**	**838 ± 262 (11)**
G112D/M230I	**25 ± 2 (20)**	**12 ± 1 (30)**	**194 ± 40 (18)**	**11 ± 0.9 (17)**	**1,341 ± 163 (17)**

a Nanomolar concentration, shown as average (standard deviation) of 3 or 4 independent experiments, each performed in triplicate. Bold indicates that the *P* value was <0.05 compared to the WT value in all independent experiments.

**FIG 4:**
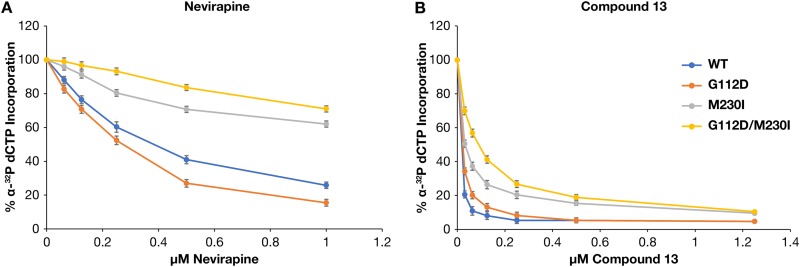
Effects of various NNRTI concentrations on DNA polymerization by WT RT and the various RT variants. As described in Materials and Methods, the value with no NNRTI present in the reaction is considered 100% activity (for all RT variants) and the other reactions are normalized to this value. Experiments were done in triplicate. (A) Reactions done in the presence of nevirapine. (B) Reactions done in the presence of compound 13.

The ability of the mutations to reduce the susceptibility of RT to NNRTIs was examined using a [α-^32^P]dCTP incorporation assay, comparing WT RT with each of the RT variants (G112D, M230I, or G112D/M230I) in the presence of NVP or compound 13. WT RT was inhibited by NVP, while G112D RT appeared to be slightly hypersensitive. The RT data were similar to the virology data, showing that M230I RT was less susceptible to NVP than WT RT and G112D/M230I RT was less susceptible than M230I RT (Fig. 4A). In our assays, compound 13 was more potent at inhibiting the polymerase activity of RT than NVP (Fig. 4B). G112D RT was also potently inhibited by compound 13. While compound 13 still affected the polymerase activity of the mutant RTs, M230I RT and the double mutant were less susceptible to inhibition than WT RT. The order of susceptibility (from least to most) is G112D/M230I > M230I > G112D > WT. M230I was selected by RPV in cell culture (5) but is rarely seen in HIV-infected individuals (14). However, the data show that while G112D has little effect on the susceptibility of RT to an NNRTI by itself, the acquisition of this mutation in the presence of M230I can reduce the susceptibility of RT to NNRTI inhibition. As noted earlier, this is a surprising result, because G112D is near the polymerase active site and is a considerable distance from M230I (>10.0 Å) (Fig. 3).

### G112D enhances HIV-1 replication in combination with M230I.

**FIG 5:**
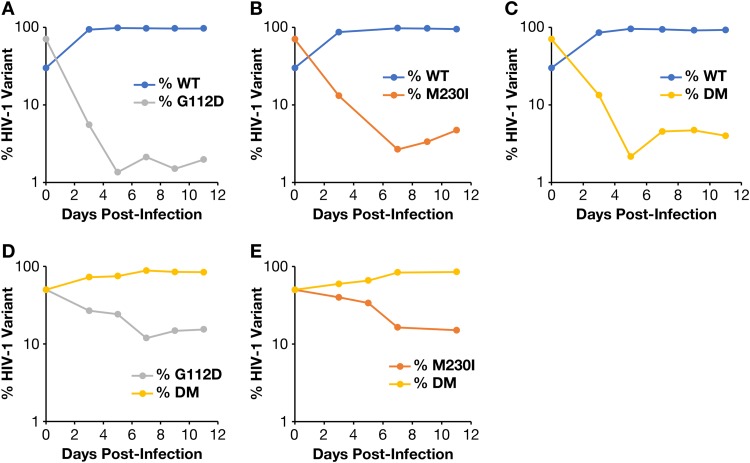
Competition assays were performed with pairs of HIV-1. HuT-R5 cells were infected with WT HIV-1 and G112D HIV-1 (A), WT HIV-1 and M230I HIV-1 (B), WT HIV-1 and G112D/M230I HIV-1 (double mutant [DM]) (C), G112D HIV-1 and G112D/M230I HIV-1 (DM) (D), or M230I HIV-1 and G112D/M230I HIV-1 (DM) (E). Viral RNA isolated from each culture at days 3, 5, 7, 9, and 11 postinfection was sequenced using Primer ID MiSeq.

As described above, the addition of G112D to the mutation M230I decreases the susceptibility of the RT to inhibition by NNRTIs. However, because some mutations can be selected if they improve the replication of an unfit virus, we performed competition experiments with HIV-1 molecular clones that encode WT RT, the G112D and M230I mutations by themselves, or both mutations in HuT-R5 cells in the absence of inhibitor. WT HIV-1 replicated substantially better than either of the single mutants (Fig. 5A and B) or the double mutant (Fig. 5C). The double mutant G112/M230I HIV-1 replicated better than either G112D HIV-1 (Fig. 5D) or M230I HIV-1 (Fig. 5E). These results suggest that G112D not only reduces the susceptibility of the M230I HIV-1 to NNRTIs but also enhances the replication of the mutant.

**FIG 6:**
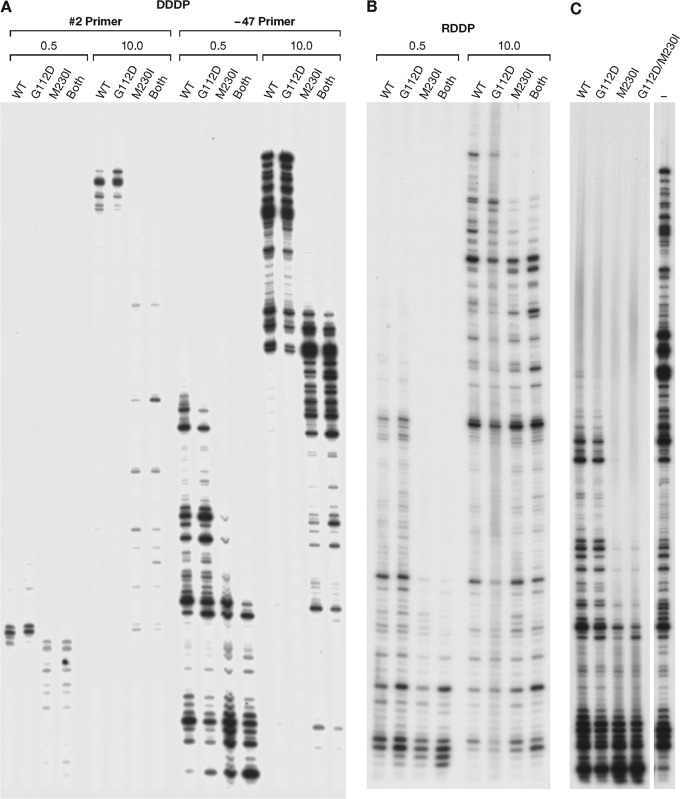
DNA-dependent DNA polymerase (DDDP), RNA-dependent DNA polymerase (RDDP), and processivity activities of the RT variants compared to the WT RT. (A) Two different primers (no. 2 and −47) were 5′ end labeled and annealed to single-stranded M13mp18 DNA in the DDDP assays. These primers were then extended by WT RT or by an RT variant in the presence of low (0.5 μM each) and high (10.0 μM each) concentrations of dNTPs. Resulting DNA products were fractionated on a 6% polyacrylamide gel. (B) A 5′-end-labeled PBS primer was annealed to an RNA template (an ∼700-nt RNA generated from a single LTR sequence) for the RDDP assay. The primer were then extended by WT RT or by an RT variant in the presence of low (0.5 μM each) and high (10.0 μM each) concentrations of dNTPs. Resulting DNA products were fractionated on a 6% polyacrylamide gel. (C) The −47 primer was 5′ end labeled and annealed to single-strand M13mp18 DNA. RT was allowed to bind to the labeled T/P. The primer extension reactions by WT RT or by an RT variant was initiated by the addition of dNTPs (final of 10.0 μM each dNTP) and the nucleic acid “cold trap,” which should have limited the extension by RT to one round. Resulting DNA products were fractionated on a 6% polyacrylamide gel. The extension of the labeled primer in the absence of the cold trap is on the far right and labeled “−.”

The polymerase activity of WT RT and the RT variants were analyzed using DNA-dependent DNA polymerase (DDDP) and RNA-dependent DNA polymerase (RDDP) assays. As described in Materials and Methods, two different DNA:DNA substrates were used for the DDDP assays, and one RNA:DNA substrate was used for the RDDP assay. To test the effects of deoxynucleoside triphosphate (dNTP) concentrations on the polymerase activity, two different dNTP concentrations (0.5 μM or 10.0 μM each dNTP) were used. We tested the ability of RT to polymerize with two related DNA:DNA substrates (primer −47 or primer no. 2 annealed to single-stranded M13mp18 DNA). With the no. 2 template/primer (T/P), the ability of G112D RT to extend a primer was similar to that of WT RT at both the low and the high dNTP concentrations (Fig. 6A). M230I had a deleterious effect on polymerization at both the low and high concentrations of dNTPs with both of the DDDP substrates. The behavior of the double mutant was similar to that of M230I RT. The mutants were also tested in an RDDP assay using a 5′-end-labeled primer binding site (PBS) primer annealed to an RNA template (see Materials and Methods) (Fig. 6B). At both dNTP concentrations, G112D RT appeared to have activity similar to that of WT RT. M230I RT again had decreased polymerase activity compared to that of WT RT. The double mutant was slightly less active than M230I RT.

Because M230I is in a position to interact with the primer strand of the T/P, it is possible that this mutation could negatively affect the stability of the binding of the M230I-containing RTs to the nucleic acid substrate. To test this idea, we used a processivity assay. Unlike the DDDP assay, in which the RTs can associate and disassociate from the T/P multiple times, the processivity assay includes an unlabeled nucleic acid “cold trap.” The various RTs are allowed to bind to the labeled nucleic acid substrate first. The reaction is initialized by the addition of dNTPs and the “cold trap.” Any RT that disassociates from the labeled T/P is more likely to bind to the cold trap, which is present in excess. Therefore, the RT is limited to one cycle of primer extension. This assay can give evidence of how well the RT remains bound to the labeled T/P. As seen in Fig. 6C, the extension products were shorter than the products made in the DDDP assay, which is the expected result, because RT is limited to a single round of extension. However, the overall results were similar to the results of the polymerase assays. The difference in the results of the two assays is not sufficient to suggest decreased nucleic acid binding ability for the M230I-containing mutants. The decreased ability to bind dNTPs and/or ability to polymerize could also explain the results.

These data indicate that while the M230I mutation had a deleterious effect on the polymerase activity *in vitro*, G112D did not help correct this deficiency. A possible explanation for the difference seen in the *in vitro* data and the virological data is that the improvement in the replication of virus carrying both mutations, compared to M230I alone, is somewhat small. Because the amount of DNA synthesized in the *in vitro* reactions is quite limited, particularly compared to the synthesis of the full-length HIV-1 genome, small differences in polymerase activity that may not be detected in biochemical assays may be detectable in cell culture assays in which the virus is actively replicating. It is also possible that the mutations could have affected the production and/or maturation of RT. The biochemical assays are done with purified RT. In the viral assays, the RT must be correctly processed from the Gag-Pol precursor. This could also play a role in the differences detected between the biochemical assays and the viral assays.

**FIG 7:**
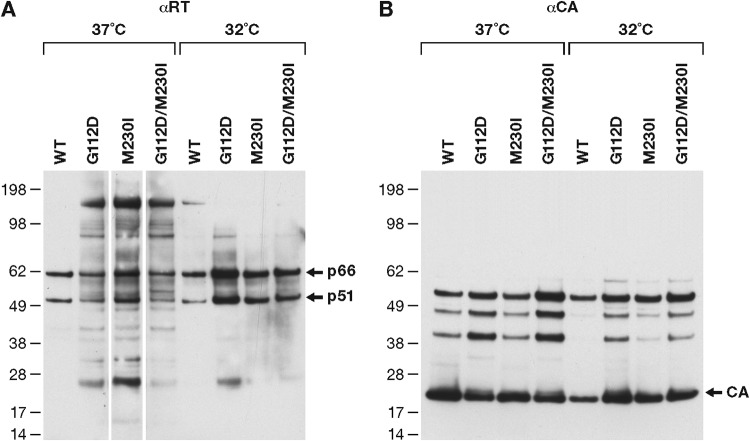
RT incorporation and processing in HIV-1 particles. The viruses are limited to a single cycle of replication. Equal amounts of WT and mutant virus (as measured by p24) were prepared from cells that were infected at either 37 or 32°C. The viruses were disrupted and were fractionated on an SDS-PAGE gel. The proteins were transferred onto a polyvinylidene difluoride (PVDF) membrane and blotted using a mixture of monoclonal antibodies that detect HIV-1 RT (A) or HIV-1 p24 CA (B). The location of the two subunits of HIV-1 RT (p66 and p51) as well as the p24 CA are marked on the right.

We previously reported that reduced levels of enzymatically active RT in virions led to hypersusceptibility of HIV-1 to azidothymidine (AZT), but not 2,3-dideoxy-3-thiacytidine (3TC), and that the results were likely due to the ability of RT to excise AZT (15). Similarly, HIV-1 with protease mutations had lower levels of mature RT in virus particles, which also led to AZT hypersensitivity (16). Thus, we assayed the amount of intact RT and relative levels of RT per virion for WT HIV-1 and the mutants. The viruses used in these drug inhibition assays are limited to a single cycle of replication (see Materials and Methods). Viruses were produced in cells incubated at 37°C or 32°C, and equal amounts of each virus, as measured by p24 enzyme-linked immunosorbent assay (ELISA), were loaded onto a Western blot that was probed with anti-RT (Fig. 7A) or anti-p24 capsid (CA) (Fig. 7B) antibodies. The mutant viruses all showed some abberant processing of Gag-Pol and/or RT; however, the double mutant was not obviously better, in terms of the correct processing of RT, than either of the single mutants (Fig. 7A). We have shown, in the past, that some mutant forms of RT that are misprocessed at 37°C are processed normally at 32°C, presumably because they are better folded at the lower temperature (17, 18). The data obtained at 32°C suggest that the amounts of RT that are made and packaged are reasonably normal and that it is the processing of the mutant Gag-Pols that is aberrant (Fig. 7A). This interpretation is supported by the fact that the processing of Gag appears to be normal (Fig. 7B). To rule out any differences in the protease, the protease coding region of the viruses that were selected in the presence of compound 13 were sequenced; no mutations were found.

### G112D and M230I affect the sensitivity of HIV-1 to NRTIs in different ways.

Because G112D is near the active site, we evaluated the effects of this mutation, with or without M230I, on the sensitivity of HIV-1 to different nucleoside analogs (Table 3). Surprisingly, all three mutants were hypersensitive to AZT, a thymidine analog, in a viral replication assay, compared to WT HIV-1. M230I HIV-1 was also hypersensitive to didanosine (ddI), an adenosine analog; to tenofovir (TFV), an adenosine nucleotide analog; and to phosphonoformic acid (PFA), a pyrophosphate analog. Both G112D and the double mutant caused hypersensitivity to PFA but had only a modest effect on susceptibility to ddI and TFV.

**TABLE 3:** WT and mutant HIV-1 susceptibility to NRTIs and PFA

Virus	EC_50_^*a*^ (fold change above WT)				
	AZT^*b*^	ddI^*c*^	FTC^*c*^	TFV^*c*^	PFA^*b*^
WT	41 ± 6 (1)	6 ± 0.5 (1)	0.5 ± 0.2 (1)	5 ± 0.8 (1)	47 ± 6 (1)
G112D	**9 ± 4 (0.2)**	**11 ± 2 (2)**	**8 ± 3 (16)**	3 ± 0.7 (1)	**8 ± 2 (0.1)**
M230I	**12 ± 6 (0.3)**	**0.8 ± 0.4 (0.2)**	**0.04 ± 0.01 (0.1)**	**1 ± 0.1 (0.3)**	**12 ± 2 (0.2)**
G112D/M230I	**7 ± 7 (0.2)**	4 ± 0.4 (1)	**2 ± 0.6 (4)**	3 ± 0.5 (1)	**5 ± 0.3 (0.1)**

a Average and standard deviation of 3 or 4 independent experiments, each performed in triplicate. Bold indicates that the *P* value was <0.05 compared to the WT value in all independent experiments.

b EC_50_ is expressed as nanomolar concentration.

c EC_50_ is expressed as micromolar concentration.

**FIG 8:**
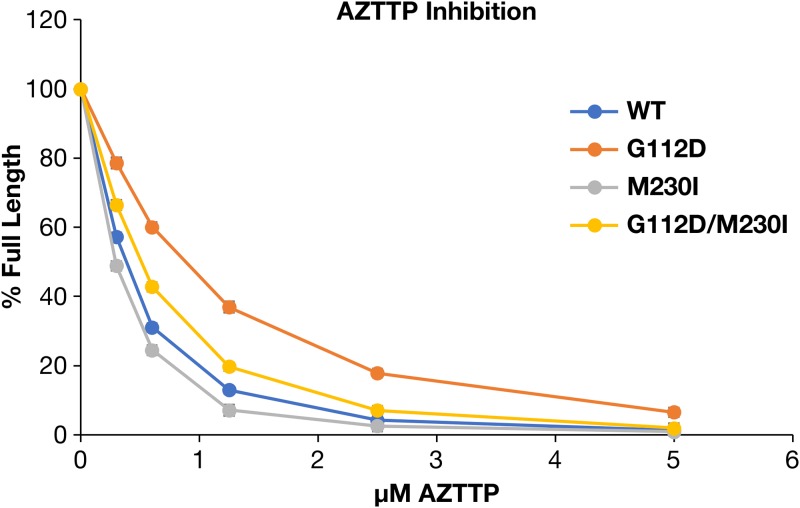
Effects of the various mutations on the ability to incorporate AZTTP into a DNA:DNA substrate. As described in Materials and Methods, the values obtained in the absence of AZTTP are considered 100% activity, and the values obtained with AZTTP in the reaction were normalized to this value.

The ability of the NRTI triphosphates (NRTITP) to inhibit the polymerase activity of the various RTs was tested on a template/primer substrate in which the 5′ end of the primer strand was labeled. The ability of either a mutant or WT RT to extend the primer to the end of the template strand (in the absence of any NRTITP) is considered 100% full length. The amount of full-length product synthesized in the presence of various concentrations of the inhibitors was normalized to this value. WT, M230I, and G112D/M230I RTs were fully sensitive to zidovudine triphosphate (AZTTP) (Fig. 8). G112D RT showed only a slight reduction in sensitivity to the inhibitor. Thus, the results seen with AZTTP in an *in vitro* polymerase assay do not explain the results seen with AZT in the viral drug susceptibility assay (Table 3), since all RTs misincorporate the analog to similar degrees.

**FIG 9:**
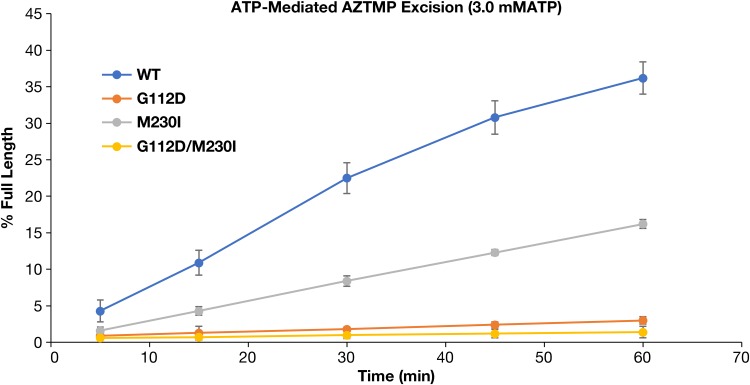
Effects of the various mutations on the ability to excise the AZTMP blocking group from the end of a primer using ATP as the pyrophosphate donor. As described in Materials and Methods, the primer strand of a DNA:DNA template/primer was blocked with AZTMP. The WT and mutant RTs were incubated with this blocked substrate in the presence of a pyrophosphate donor (ATP) and dNTPs.

The major pathway for AZT resistance involves the selective excision of the AZT monophosphate (AZTMP) from the end of the primer after AZTTP has been incorporated. This excision reaction is closely related to the polymerase reaction run in reverse, but it uses ATP as the pyrophosphate donor. The excision reaction frees the 3′ end of the primer strand and produces a dinucleoside tetraphosphate (19–21). WT RT is able to excise AZTMP using ATP as the pyrophosphate donor to a limited degree (22, 23). It is possible that G112D and/or M230I could reduce this intrinsic excision ability, which would explain the hypersensitivity of the mutant viruses to AZT. Both G112D and G112D/M230I RTs showed only very limited excision ability using ATP (Fig. 9) as the pyrophosphate donor. M230I RT was better at excision but was still less excision proficient than WT RT. These results suggest that the hypersensitivity seen in virology assays is probably caused by the fact that both mutations, especially G112D, reduce the ability of RT to excise AZTMP.

The viruses carrying the single and double mutations had distinct phenotypes in the presence of 2′,3′-dideoxy-5-fluoro-3′-thiacytidine (FTC) (Table 3). G112D reduced the susceptibility of the virus to FTC 16-fold, while M230I conferred hypersensitivity. The double mutant had an intermediate phenotype: a 4-fold loss of susceptibility. Addition of the FTC resistance mutation M184V to M230I (M184V/M230I HIV-1) produced the expected loss of susceptibility to FTC but did not affect the susceptibility of the virus to AZT or RPV (data not shown). M184V causes reduced residual excision by RT (24–27). Because the M230I reduces the ability of RT to excise, it is not surprising that AZT susceptibility is not further reduced by the M184V/M230I double mutant. G112D/M184V HIV-1 was not infectious and could not be characterized in cell culture assays.

**FIG 10:**
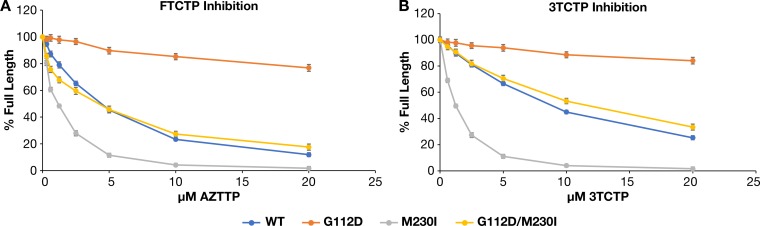
Effects of the various mutations on the ability to incorporate FTCTP (A) or 3TCTP (B) into a DNA:DNA substrate. As described in Materials and Methods, the values obtained in the absence of FTCTP or 3TCTP is considered 100% activity, and the other values (with FTCTP or 3TCTP in the reaction) are normalized to this value.

WT RT and G112D/M230I RT showed similar sensitivities to FTC triphosphate (FTCTP) (Fig. 10A) in biochemical assays; this result was confirmed by the results obtained in the virology assays. However, G112D RT was relatively insensitive to FTCTP and M230I RT was hypersensitive. FTCTP has a fluorine group on the cytosine nucleobase, which raises the question of whether this group affected the susceptibility of the mutant RTs. We tested 3TC triphosphate (3TCTP), which is similar to FTCTP in its overall structure but lacks the fluorine group (Fig. 10B). The susceptibility of the mutants to 3TCTP was similar to that to FTCTP, suggesting that it is the oxythiolane ring, which is present in both drugs, that is responsible. The fact that the M230I mutation affects the incorporation of both FTCTP and 3TCTP, taken together with the fact that G112D acts to compensate for the effects of the M230I mutation on the incorporation of these analogs, provides additional support for the proposal, made earlier, that M230I affects the positioning of the nucleic acid substrate at the active site.

## DISCUSSION

Due to the high mutation rate of HIV-1, drug resistance mutations can arise in infected individuals, which can lead to resistance to an entire class of antiretroviral drugs. Thus, new compounds that are effective against drug-resistant mutants are needed. Analogs of the NNRTI RPV were developed and showed efficacy *in vitro* to known NNRTI resistance mutations (11). Compound 13 was used to select for mutations in HIV-1 RT, and in parallel, as a control, selection was also performed with RPV. In the selection, resistance to RPV appeared first; moreover, in terms of the fold change in the levels of the compounds that would still permit replication of the selected virus, compound 13 was obviously better than RPV (8-fold versus 128-fold, respectively). These results suggest that it is more difficult for HIV-1 to become resistant to compound 13 in cell culture. Interestingly, the viral mutants selected with compound 13 had two unusual mutations, M230I and G112D, which seem to work together. This pair of mutations caused changes in the susceptibility of RT to both NNRTIs and NRTIs.

**FIG 11:**
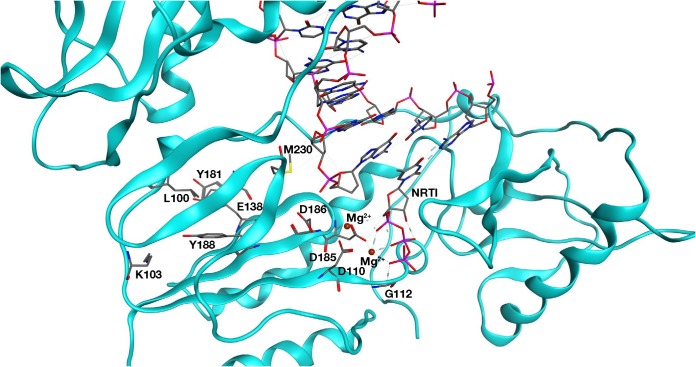
Overview of the RT polymerase active site structure, indicating the position of the nucleic acid and the G112 and M230 positions. The RT ribbon backbone (in cyan) is modeled with the nucleic acid (no ribbon backbone) and an NRTI bound in the RT DNA polymerase active site (aspartic residues labeled). Residues G112 and M230 are shown in relation to the bound nucleic acid and are labeled. The NNRTI binding pocket (signature residues that comprise the binding pocket are labeled) sits behind the RT DNA polymerase active site. Carbon atoms are gray, nitrogen atoms blue, oxygen atoms red, sulfur atoms yellow, and phosphates pink.

These two mutations are approximately 10 to 12 Å apart in the structure of RT (Fig. 3 and 11). M230 is in the periphery of the hydrophobic pocket that binds NNRTIs, and M230I is a known resistance mutation that has been selected by RPV in cell culture (5, 14). When RPV is bound to HIV-1 RT, M230I is near the cyanovinyl group on the longer side chain of RPV (Fig. 3) (4). As described above, M230 is also near the “primer grip” region of the polymerase domain, which interacts with the primer strand and helps to align the 3′ end of the primer strand with the polymerase active site (Fig. 11) (3). However, RT containing the M230I mutation has not been extensively studied biochemically, and whether this mutation affects the positioning of the primer strand is not known. One study showed that M230I could compensate for the deleterious effects of the Y115W mutation in HIV-1 RT, suggesting that the M230I mutation affected the primer grip (28, 29). Our results suggest that by itself, the M230I mutation has a deleterious effect on the ability of RT to extend a primer. It is likely that this is also a result of the effects of M230I on the primer grip region of RT. Because the 3′ end of the primer strand and corresponding portion of the template strand are an integral part of the polymerase active site, M230I could affect the positioning of the end of the primer strand at the polymerase active site. Because the end of the primer strand is optimally positioned in WT RT, a change in positioning of the primer could decrease the polymerase activity of the RT.

Mutations at position G112 have rarely been detected in HIV-1 RT. G112 is highly conserved, and this amino acid lies in a region that is crucial for polymerase activity. D110 is one of the polymerase active site residues (Fig. 3 and 11), and the Y115 and F116 residues are located under the sugar ring of incoming dNTPs. Mutations at Y115 can alter the fidelity and activity of the RT (28, 29), and Y115 appears to be a “gatekeeper” in the polymerase active site that prevents NTPs from being properly bound and incorporated (30). Mutations at the analogous position in Moloney murine leukemia virus (MMLV) RT (F155) have a similar gatekeeper effect (31, 32). G112 has no direct interactions with the NNRTI binding pocket (Fig. 3 and 11). However, other experimental NNRTIs have selected two mutations at this position, or its equivalent in HIV-2 RT. In HIV-2 RT, whose structure is closely related to the structure of HIV-1 RT, a phenylmethylthiazolylthiourea (PETT) derivative, MSK-076, selected for the mutations A101P and G112E (33). These resistance mutations were mapped on the structure of HIV-2 RT, and A101P is near the NNRTI-binding site of HIV-1 RT. In HIV-2 RT, G112E is in a position that is equivalent to G112 in HIV-1 RT (33). The NNRTIs VRX-329747 and VRX-413638 selected for multiple mutations in HIV-1 RT, including G112S (34). It is unclear from those studies how G112 amino acid substitutions affected either susceptibility to NNRTIs or the polymerase activity of RT.

While G112D alone had little effect on susceptibility to NNRTIs, M230I reduced susceptibility to all of the NNRTIs tested. This is not surprising because this mutation is located in the NNRTI binding pocket and the mutated amino acid can interact directly with a bound NNRTI. However, combining G112D with M230I further reduced the susceptibility of RT to NNRTIs. It is not immediately apparent from its location, near the polymerase active site, how G112D is able to interact with M230I to reduce the susceptibility of RT to NNRTIs. Because M230I alters the organization of the polymerase active site through its interactions with the primer strand, it has two roles: (i) it interacts directly with a bound NNRTI and (ii) it interacts with, and affects, the positioning of the nucleic acid. Therefore, it is probable that G112D and M230I interact through their effects on the bound nucleic acid. The effect of the G112D mutation on NNRTI susceptibility is relatively modest and might affect the bound nucleic acid. Although a bound NNRTI does not prevent the binding of nucleic acid to RT, it does alter the trajectory on the nucleic acid, pushing the 3′ end of the primer away from the active site (1). We propose that the G112D mutation opposes the displacement of the end of the viral DNA from the active site. This could, in turn, affect NNRTI binding because it would make it more difficult for an incoming NNRTI to enter the hydrophobic pocket and displace the bound nucleic acid.

Because the M230I and G112D mutations alter the polymerase active site, we considered the possibility that one, or both, of these mutations could affect the incorporation of, or susceptibility to, NRTITPs, especially those that have structural features that lie outside the substrate envelope of a normal dNTP (e.g., AZTTP or FTCTP). The virology data indicate that the mutant viruses are hypersensitive to AZT, which could mean that AZTTP is better able to compete with dTTP in mutant RTs. However, when we compared the ability of purified WT and mutant HIV-1 RTs to incorporate AZTTP in biochemical assays, none of the mutants incorporated AZTTP better than the WT. At most, G112D RT showed a slight reduction in susceptibility to AZTTP. The explanation for this apparent discrepancy lies in the most common mechanism of AZT resistance (19, 23, 35, 36). Most NRTI resistance mutations interfere with the incorporation of the NRTITP, either directly (e.g., M184V/I, which causes steric hindrance involving the oxathiolane ring of 3TCTP or FTCTP) or indirectly (e.g., K65R altering the ability of the RT to bind tenofovir relative to dATP). In contrast, the most common AZT resistance mutations affect not the incorporation of AZTTP into the elongating primer strand but rather the ATP-mediated excision of AZTMP from the end of the viral DNA after it has been incorporated. The primary resistance mutations to AZT are T215F/Y and/or K70R. These mutations improve the binding of the ATP, which acts as a pyrophosphate donor in the excision of the incorporated AZTMP from the end of the primer strand, producing a dinucleoside tetraphosphate (AppppAZT) and freeing the 3′ end of the primer for further elongation. WT RT can excise AZTMP, but ATP binding is weak, and as a consequence, WT RT is much less proficient at excision than AZT-resistant mutants (23). In this study, we showed that the G112D, M230I, and G112D/M230I mutations significantly decrease the ability of RT to excise AZTMP using ATP. M230I has the smallest effect on excision, which agrees with the data obtained in the virology assays. Some mutations in the RT active site, including M184I/V, cause AZT hypersensitivity by reducing the ability of RT to excise AZTMP (24, 27). This suggests that the G112D could also reduce AZTMP excision. Normally, an incoming dNTP binds to the nucleotide binding site (N site; for background information on the excision reaction, see references 21 and 36). Immediately after a dNMP is added to the 3′ end of the primer strand, the 3′-OH group is located at the N site. Normally, translocation moves the primer back so that the newly incorporated dNMP is located at the P site. However, for an AZTMP at the end of the primer strand to be excised, the end of the primer must remain in the N site, where AZTMP excision can be catalyzed by the polymerase active site. The long azido group of AZTMP interferes with the translocation of the end of the primer from the N to the P site, which is why AZTMP is excised much more efficiently than other NRTIMPs. If the G112D mutation alters the active site in a way that would permit the translocation of an AZTMP at the end of the primer from the N to the P site, excision by RT would be reduced, and this would explain why the mutants are hypersensitive to AZT.

The mutations also affect the sensitivity of HIV-1 to FTC and the related drug 3TC. Both drugs have an unusual oxathiolane pseudosugar ring instead of the deoxyribose sugar ring that is present in normal dNTPs. The oxathiolane ring of both of these compounds is in the l, as opposed to the normal d, configuration. In addition, there is a sulfur that is part of the ring structure. As a consequence, a portion of the oxathiolane ring lies outside of the substrate envelope that is defined by the binding of normal dNTPs that have a deoxyribose as the sugar. The portions of the oxathiolane ring that lie outside the envelope defined by the bound deoxyribose allow mutant forms of RT, particularly M184V/I RT, to distinguish between the normal substrate dCTP and the analogs FTCTP and 3TCTP. We asked whether the G112D and M230I mutations affected the incorporation of FTCTP. G112D causes a reduction in both FTCTP and 3TCTP incorporation into DNA. It is likely that the altered polymerase active site interferes with ability of the oxathiolane ring of the triphosphate form of the analogs to bind in a position that is compatible with their incorporation. In contrast, M230I causes hypersensitivity to FTCTP and 3TCTP, which indicates that both of these analogs are better able to compete with dCTP for binding and incorporation by the M230I mutant RT. The most likely explanation is that the alterations in the trajectory of the nucleic acid substrate caused by M230I affect the polymerase active site in a way that allows FTCP and 3TCTP to be incorporated more efficiently. In support of this idea, the double mutant had an intermediate phenotype, suggesting that the opposite effects of the two individual mutations are compensatory in terms of their effects on the ability of RT to incorporate 3TCTP and FTCTP. This strengthens the argument that the effects of the two mutations, G112D and M230I, intersect at the active site and involve the positioning of the nucleic acid substrate.

In summary, compound 13 selected a pair of amino acid substitutions that, while distant from each other in RT, interact in ways that affect the susceptibility of HIV-1 to both NNRTIs and NRTIs. M230I can affect NNRTI susceptibility by altering the NNRTI binding pocket directly. M230I can also affect the binding of nucleic acid by altering the primer grip region, which indirectly affects the structure of the polymerase active site and NRTI susceptibility. Conversely, G112D, which is quite near the polymerase active site, can affect NRTI susceptibility directly and NNRTI susceptibility indirectly. These mutations confer resistance to compound 13 and all other NNRTIs we tested, but the mutations negatively affect polymerase activity. G112D by itself has no measurable effects on the incorporation of normal dNTPs. However, its effects on both the susceptibility of RT to both NNRTIs and NRTIs in the presence of the M230I mutation point to the importance of the nucleic acid component in the structure and function of the polymerase active site, not only for normal polymerization but also for inhibition by anti-RT drugs, including NNRTIs.

We suggest that the unusual mutation in HIV-1 RT selected by compound 13 in culture is a good sign in terms of its possible clinical utility. Although the increased use of antiretroviral therapy globally is a very promising development, it carries with it an increase in the worldwide burden of drug-resistant HIV-1 mutants. NNRTIs in general, and RPV in particular, are an important component of the drug combinations that are in general use. Having new NNRTIs that select for a different spectrum of resistance mutations could add to the armamentarium that can be used in the global effort to treat HIV-1 infections. It would be even better if some of the mutations these new compounds selected for would enhance the susceptibility of the virus to other drugs that target the same enzyme.

## MATERIALS AND METHODS

### Cell lines and RT inhibitors.

293T and TZM-bl cells were cultured in Dulbecco’s modified Eagle's medium (Thermo Fisher Scientific) supplemented with 10% fetal bovine serum (FBS; Atlanta Biologicals) and 100 U/ml of penicillin, 100 μg/ml of streptomycin, and 0.292 mg/ml of l-glutamine (P-S-G; Thermo Fisher Scientific). GHOST-R3/X4/R5 cells (37) were maintained in the medium described above with the addition of 100 μg/ml of G418 (Thermo Fisher Scientific), 100 μg/ml of hygromycin (Thermo Fisher Scientific), and 0.5 μg/ml of puromycin (EMD Millipore). HuT-R5 cells (38) were cultured in RPMI 1640 medium (Invitrogen) supplemented with 10% FBS–P-S-G, 500 μg/ml of G418, and 0.5 μg/ml of puromycin.

Rilpivirine and compound 13 were synthesized as previously described (11). Nevirapine (NVP), efavirenz (EFV), etravirine (ETR), zidovudine (AZT), didanosine (ddI), emtracitabine (FTC), and tenofovir (TFV) were obtained from the AIDS Reagent Program, Division of AIDS, NIH. Phosphonoformic acid (PFA) was a gift from John Mellors.

### Virus production.

Replication-competent HIV-1_xxLAI_ (39) was used in all cell culture assays and is referred to hereafter as HIV-1. RT mutations were made in a plasmid carrying the viral DNA using a QuikChange II XL site-directed mutagenesis kit (Agilent Technologies). Viruses were generated by transfection of 293T cells using Lipofectamine 2000 (Thermo Fisher Scientific), and titers were determined on GHOST-R3/X4/R5 cells as previously described (40).

### HIV-1 selection with NNRTIs.

HuT-R5 cells were infected with HIV-1 at a multiplicity of infection (MOI) of 0.05 and cultured in medium containing inhibitors at a concentration 2- or 4-fold above the reported 50% effective concentration (EC_50_) (11). Cells were split every 2 to 3 days. Cell aliquots were pelleted and stored at −80°C. Supernatants were filtered through a 0.45-mm syringe filter and stored at −80°C. When cytopathic effects were noted in the cultures, inhibitor concentrations were increased 2-fold.

Viral RNA was isolated from culture supernatants with the RNeasy minikit (Qiagen). cDNA was generated using random hexamer primers with the SuperScript III first-strand synthesis kit (Thermo Fisher Scientific). Cellular DNA was isolated from infected cells using the QIAamp DNA minikit (Qiagen). The RT coding region from cDNA or cellular DNA was amplified using Platinum PCR supermix (Thermo Fisher Scientific) with primers LAI-RTF (5′-TTTGCCAGGAAGATGGAAAC-3′) and LAI-RTR (5′-TCACTAGCCATTGCTCTCCA-3′). PCR conditions were 94°C for 2 min, 35 cycles of 94°C for 30 s, 52°C for 30 s, and 72°C for 2 min, and 72°C for 5 min. Amplified PCR products were sequenced directly or TA cloned using the pCR 2.1-TOPO TA cloning kit (Thermo Fisher Scientific) and sequenced.

### HIV-1 competition assays.

HuT-R5 cells were infected with two viruses at a 30:70 (WT/mutant) or 50:50 (mutant/mutant) ratio with a total MOI of 0.004. Beginning at day 3, cultures were split every 2 days until day 11. Virus was pelleted from 500 μl of culture supernatant by centrifugation at 13,000 rpm for 60 min at 4°C. RNA was isolated from each sample using the RNeasy minikit (Qiagen).

Primer ID MiSeq was used to sequence part of the RT coding region of each sample using primers and methods described previously (41). Input RNA used for each sample was 20 ng. Sequence analysis was performed using a custom pipeline (https://tcs-dr-dept-tcs.cloudapps.unc.edu/), as previously described (42). The number of paired-end reads from each sample ranged from 359 to 18,597. A custom computer script was written to align the sequences and to quantify each codon representing amino acids 112 and 230 of RT. The relative abundance of each mutant was calculated by dividing the number of mutant sequences by the total number of consensus sequences at each time point. Unexpected variants were present at low levels (<0.9% of the total sequences in each sample).

### HIV-1 inhibitor susceptibility assays.

Susceptibility assays with RT inhibitors were performed in TZM-bl cells as previously described (43). Briefly, cells were incubated in the absence of any compound or in different concentrations of the individual compounds and infected with each virus. Infectivity was measured by luciferase expression in the cells, with uninfected cells set as background and infections in the absence of inhibitor set at 100% infection. Each experiment was performed in triplicate and each experiment was performed 3 or 4 times. EC_50_ values were calculated in Prism (GraphPad) using nonlinear regression for each experiment. Values are reported as the average EC_50_ values from 3 or 4 experiments with standard deviations. The extra sum-of-squares F test was used for comparison of EC_50_ values for each experiment. Differences in EC_50_ values between WT and mutant viruses were considered statistically significant if the *P* value was <0.05 in all independent experiments.

### Western blot analysis of HIV-1 proteins.

293T cells were transfected with HIV-1 proviral vector pNLNgoMIVR^-^Δenv.LUC (44) and pHCMV-g (vesicular stomatitis virus G protein [VSV-G] expression vector obtained from Jane Burns, University of California at San Diego) using calcium phosphate. Cells were grown in medium containing 2% FBS at 37 or 32°C. After 48 h, cell supernatants were collected, clarified by low-speed centrifugation, and filtered through a 0.45-μm-pore-size filter. Viruses were concentrated using Vivaspin concentrators (Sartorius) according to the manufacturer’s protocol.

The amount of p24 in the supernatant was determined using an HIV-1 p24 ELISA kit (ExpressBio). Equal amounts of virus, as measured by p24, were pelleted through a 20% sucrose cushion at 25,000 × *g* for 35 min. Viral pellets were resuspended in 1× NuPage SDS sample buffer (Invitrogen) with 10 mM dithiothreitol, heated at 65°C for 5 min, fractionated on a NuPage 4% to 12% bis-Tris gel (Invitrogen), and wet-blot transferred onto Invitrolon-ECL polyvinylidene difluoride (PVDF) (Invitrogen). The blot was blocked in 5% milk and RT was detected using a mix of monoclonal antibodies 19, 21, 42, 48, and 50 (45), followed by a secondary antibody (goat anti-mouse; KPL). The blots were developed in Femto detection solution (Pierce) and visualized on film. The blot was stripped in Restore Plus Western blot stripping buffer (Pierce) and probed with polyclonal anti-p24 antibody (kindly provided by David Ott, National Cancer Institute).

### RT polymerase assays.

The expression and purification of HIV-1 RT heterodimers have been described previously (22). The G112D and M230I mutations were introduced into the p66 coding region using the Multi-Quik change kit (Agilent Technologies) as per the manufacturer’s instructions.

Two templates/primers (T/P) were used for the DNA-dependent DNA polymerase (DDDP) assay. For the first T/P, a synthetic DNA oligonucleotide designated −47 (5′-CGCCAGGGTTTTCCCAGTCACGAC-3′) (Integrated DNA Technologies) was 5′ end labeled using [γ-^32^P]ATP and T4 polynucleotide kinase. The name −47 comes from its location on the M13mp18 DNA sequence (New England BioLabs [NEB]). The oligonucleotide was purified by passage through Centri-Spin 10 columns (Princeton Separations) and then annealed to the single-stranded M13mp18 DNA (NEB) by heating then slow cooling, to generate the DNA:DNA template/primer. This T/P has been described previously (22). The second DNA:DNA template/primer was generated using the same protocol. Primer no. 2 (5′-CACCGCCTGCAACAGTGCC-3′) was 5′ end labeled as described above and then annealed to M13mp18 DNA. For the RNA-dependent DNA polymerase (RDDP) assay, an RNA template was generated using a clone containing a T7 RNA polymerase promoter, the HIV-1 polypurine tract (PPT), one copy of the HIV-1 long terminal repeat (LTR; U3, R, and U5), and the HIV-1 primer binding site. The clone was linearized using NotI, which cleaves 3′ of the primer binding site (PBS). An RNA transcript that contains the HIV-1 PPT, the LTR, and PBS (sense strand) was synthesized using the T7 MEGAScript kit (Ambion). A synthetic DNA oligonucleotide complementary to the primer binding site sequence (5′-GTCCCTGTTCGGGCGCCA-3′) (Integrated DNA Technologies) was 5′ end labeled and then annealed to the RNA template as described above.

The labeled RNA template/DNA primer or DNA template/DNA primer combination was resuspended in a mixture containing 25 mM Tris (pH 8.0), 75 mM KCl, 8.0 mM MgCl_2_, 100.0 μg of bovine serum albumin (BSA) per ml, and 10.0 mM CHAPS {3-[(3-cholamidopropyl)-dimethylammonio]-1-propanesulfonate}. dNTPs were present at either 0.5 μM each or 10.0 μM each. The reactions were initiated by the addition of the wild-type (WT) RT or one of the RT mutants. The reactions were allowed to proceed at 37°C for 10 min and then halted by extraction with phenol-chloroform. The nucleic acids were precipitated by the addition of 2 volumes of ethanol. The recovered nucleic acids were fractionated by electrophoresis on a 6.0% polyacrylamide gel and dried, and the gel was autoradiographed.

The processivity assay is similar to the DDDP assay. The RT is allowed to bind to the ^32^P-end-labeled T/P in 1× reaction buffer (25 mM Tris [pH 8.0], 75 mM KCl, 8.0 mM MgCl_2_, 100.0 μg of BSA per ml, and 10.0 mM CHAPS). The reaction is initiated by the addition of dNTPs (1× = 10.0 μM each dNTP) and an unlabeled poly(rC) · oligo(dG) nucleic acid “cold trap” (0.6 U/μl) (Pharmacia). The cold trap is a long (300- to 500-nucleotide [nt]) rC strand with several oligo(dG) (15–17, 22, 23) primers annealed to it. Unlike in the DDDP assay, in which the RT can disassociate from the labeled nucleic acid and then potentially rebind, the cold trap sequesters any RT that has become unbound from the labeled T/P. Hence, extension is limited to just one round of RT binding.

### *In vitro* inhibition assays. (i) NNRTI inhibition assay.

The nonnucleoside reverse transcriptase inhibitor (NNRTI) inhibition assay was similar to the polymerase assay described above. However, the DNA primer was not 5′ end labeled. The unlabeled primer was annealed to single-stranded M13mp18 DNA as described above. The annealed template/primer was suspended in a mixture of 25 mM Tris (pH 8.0), 75 mM KCl, 8.0 mM MgCl_2_, 100.0 μg of BSA per ml, 10.0 mM CHAPS, and 10.0 μM (each) dATP, dTTP, dGTP, and dCTP. The radioactive triphosphate [α-^32^P]dCTP was included in the reaction mixture and acted as a tracer. The reactions also contained various concentrations of the NNRTI as shown in the figures. The reactions were initiated by the addition of WT RT or an RT variant and allowed to proceed for 30 min at 37°C. The reactions were halted by the addition of 200 μl of 10.0 mg/ml of sheared/denatured salmon sperm DNA, 200 μl of 0.2 M NaPP_i_ (Sigma), and 3.0 ml of 10% trichloroacetic acid (TCA). The precipitated DNA was captured on Whatman GF/C glass filters by suction filtration and the filters were added to scintillation vials. After addition of scintillation fluid, the filters were counted. The number of counts in the absence of NNRTI was considered 100% activity, and the other counts obtained at the other NNRTI concentrations were normalized to this value.

### (ii) NRTITP inhibition assay.

A synthetic DNA oligonucleotide (5′-CAGGTCACTGTTCGAGCACCA-3′; Integrated DNA Technologies) was 5′ end labeled and then annealed to the DNA template (5′-GCGCAGTGTAGACAATCCCTAGCTATGGTGCTCGAACAGTGACCTG-3′). The annealed template/primer was suspended in a mixture of 25 mM Tris (pH 8.0), 75 mM KCl, 8.0 mM MgCl_2_, 100.0 μg of BSA per ml, 10.0 mM CHAPS, and 10.0 μM (each) dATP, dTTP, dGTP, and dCTP. The reactions also included various concentrations of the triphosphate form of the analog (nucleoside reverse transcriptase inhibitor triphosphate [NRTITP]) as shown in the figures. The reactions were initiated by the addition of WT RT or a mutant RT and allowed to proceed for 10 min at 37°C. The nucleic acids were purified by extraction with phenol-chloroform and then ethanol precipitated. After centrifugation, the samples were resuspended in gel loading buffer II (Ambion) and then fractionated on a 15% polyacrylamide sequencing gel. The total amount of template/primer (analog-blocked products and full-length products) and the amount of full-length product (extended to the end of the template) were determined by using a PhosphorImager. The amount of full-length product in the absence of any NRTITP was considered 100% activity. The amount of full-length product produced with the NRTITPs was normalized to that value. The percentage of full-length product was calculated by determining the amount of primer that was extended to the end of the template and dividing this amount by the amount of all extension products (full-length and NRTIMP-blocked primers).

### Excision assays.

The ATP-dependent pyrophosphorolysis assay was similar to the polymerization assay described above. A synthetic DNA oligonucleotide (5′-CAGGTCACTGTTCGAGCACCA-3′; Integrated DNA Technologies) was 5′ end labeled and then annealed to the DNA template (5′-GCGCAGTGTAGACAATCCCTAGCTATGGTGCTCGAACAGTGACCTG-3′). The 3′-OH at the end of the primer was blocked by the addition of AZTTP, using WT RT. The underlined letter in the template indicates the position where the AZTTP was added onto the primer. After purification, the template/primer was incubated with HIV-1 RT or a mutant RT in a mixture of 50.0 mM HEPES (pH 7.5), 75 mM KCl, 16.0 mM MgCl_2_, 100.0 μg of BSA per ml, 10.0 mM CHAPS, 10.0 μM (each) dATP, dTTP, dGTP, and dCTP, and 3.0 mM ATP for the lengths of time indicated in the figure. ATP was obtained from ChemCyte. The reactions were halted by phenol-chloroform extraction: the salts and nucleosides/nucleotides were removed by passage through a Centri-Spin 10 column (Princeton Separations), and the template/primer was precipitated by the addition of ethanol. The products were fractionated on a 15% polyacrylamide sequencing gel. The total amount of template/primer (blocked and unextended plus deblocked and extended) and the amount of full-length product (deblocked and extended to the end of the template) were determined using a PhosphorImager.
